# Supplementary material for: Effectiveness and Safety of Ayurvedic Medicines in Type 2 Diabetes Mellitus Management: A Systematic Review and Meta-Analysis
Source: Front Pharmacol. 2022 Jun 8;13:821810. doi: 10.3389/fphar.2022.821810 (PMC9213670; doi:10.3389/fphar.2022.821810)
Supplement: Supplementary file 1 [file DataSheet1.zip › Supplementary Material/Forest and Funnel Plots/Gynostemma pentaphyllum/Body weight.pdf]

Gynostemma pentaphyllum - Body weight

	Intervention			Comparator			Mean Diff.	Weight
Study	N	Mean	SD	N	Mean	SD	with 95% CI	(%)
Huyen 2010	12	60.50	9.50	12	57.00	10.50	3.50 [-4.51, 11.51]	43.06
Huyen 2012	12	55.10	6.50	13	54.80	10.60	0.30 [-6.67, 7.27]	56.94
**Overall**							1.68 [-3.58, 6.93]	

Heterogeneity: $\tau^2 = 0.00$, $I^2 = 0.00\%$, $H^2 = 1.00$

Test of $\theta_i = \theta_j$: Q(1) = 0.35, p = 0.55

Test of $\theta = 0$: z = 0.63, p = 0.53

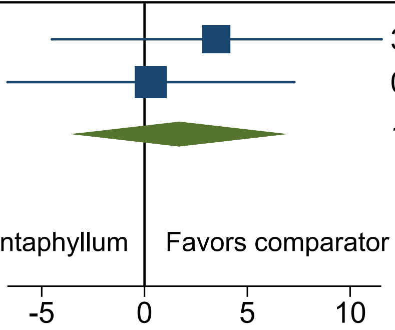

Random-effects REML model
